# ﻿Molecular and morphological identification of larvae of Carangidae (Teleostei, Carangiformes) species from southern Gulf of California

**DOI:** 10.3897/zookeys.1212.118644

**Published:** 2024-09-16

**Authors:** Claudia A. Silva-Segundo, René Funes-Rodríguez, Eduardo Anaya-Godínez, Jaime Gómez-Gutiérrez

**Affiliations:** 1 Instituto Politécnico Nacional, Centro Interdisciplinario de Ciencias Marinas, Departamento de Plancton y Ecología Marina, Av. IPN s/n, CP 23096, La Paz, Baja California Sur, Mexico Instituto Politécnico Nacional, Centro Interdisciplinario de Ciencias Marinas La Paz Mexico; 2 Departamento Académico de Ingeniería en Pesquerías, Universidad Autónoma de Baja California Sur, Carretera al Sur Km 5.5, CP 23088, La Paz, Baja California Sur, Mexico Universidad Autónoma de Baja California Sur La Paz Mexico

**Keywords:** *Caranx*, COI, *Decapterus*, early larval stages, marine reserve, Mexico, sibling species

## Abstract

The description of diagnostic morphological characters and DNA barcoding of fish larvae from nine species of the carangid family are provided from specimens collected during a weekly zooplankton time-series (2016–2017) at Cabo Pulmo National Park, Gulf of California, Mexico. Five nominal species (*Caranxsexfasciatus*, *C.caballus*, *Naucratesductor*, *Selarcrumenophthalmus*, and *Seleneperuviana*) and three morphotypes of *Decapterus* spp. and one of *Caranx* spp. were identified and separated based on morphological, meristic, and pigmentary diagnostic characters. All larvae were genetically sequenced for a fragment of the cytochrome c oxidase subunit I mitochondrial gene. Sequences of larval *Caranx* and *Decapterus* showed high genetic similarity (> 99%), low intraspecific divergence (< 1%), and an interspecific divergence between 6% and 11%, allowing the discrimination of diagnostic pigmentation patterns of fish larvae among three sibling species from each genus: *Caranx* (*C.caballus*, *C.caninus*, and *C.sexfasciatus*) and *Decapterus* (*D.macarellus*, *D.macrosoma*, and *D.muroadsi*). DNA barcoding supported the presence of *Caranxcaballus*, *C.caninus*, *C.sexfasciatus*, *Decapterusmacarellus*, *D.muroadsi*, *Selarcrumenophthalmus*, and *Seleneperuviana*, and for the first time *Naucratesductor* and *D.macrosoma* at the CPNP. Abundance of these nine species (confirmed molecularly) was estimated throughout the 2016–2017 weekly time series. *Decapterusmacarellus* and *Caranxcaninus* were the most abundant species. The morphological and molecular taxonomic methods allowed us to infer the species number and abundance of these commercial species at the CPNP to improve conservation in protected areas and fishery management.

## ﻿Introduction

Rapid, cost-effective, and precise identification of species of fish larvae facilitates better resolution to the analysis of ichthyoplankton communities (diversity, abundance, and distribution patterns). Despite the monumental taxonomic effort of fish larval description keys for fish species identification in the Eastern Pacific and other regions of the world (Moser 1996; Watson et al. 1996; Beltrán-León and Ríos-Herrera 2000; Richards 2005), there is still a high percentage of species without available ontogenetic larval descriptions relative to the fish diversity for the Mexican Pacific and Gulf of California (Kendall and Matarese 1994). The family Carangidae is highly diverse and includes several species that are targets of relevant artisanal fisheries that provide socio-economical resources to local people. At least 33 nominal species of Carangidae have been reported in the Eastern Pacific based on juvenile and adult specimens (Smith-Vaniz 1995). From them, only 18 species have had their larval stages described (Watson et al. 1996; Beltrán-León and Ríos-Herrera 2000). This taxonomic information has allowed the publication of several ichthyoplankton checklists from different regions of the Mexican Pacific, including the Gulf of California. However, several taxa from this region have frequently been identified only to the genus level (i.e., *Caranx* spp., *Decapterus* spp., *Oligoplites* spp., *Selene* spp., and *Seriola* spp.; Aceves-Medina et al. 2003; González-Armas et al. 2008; Silva-Segundo et al. 2008; Avendaño-Ibarra et al. 2014).

Five nominal species of *Caranx* genus have been reported in the Mexican Pacific, including the Gulf of California: *Caranxsexfasciatus* Quoy & Gaimard, 1825; *Caranxmelampygus* Cuvier, 1833; *Caranxlugubris* Poey, 1860; *Caranxcaninus* Günther, 1867; and *Caranxcaballus* Günther, 1868 (Smith-Vaniz 1995; Allen and Robertson 1998; Froese and Pauly 2000). Sumida et al. (1985) reported morphological and meristic descriptions of fish larvae of *Caranxcaballus* and *Caranxsexfasciatus*. Those larval descriptions were later evaluated using molecular DNA barcoding methods that concluded that the main diagnostic morphological characters originally assigned to *C.sexfasciatus* were larvae of *C.caninus*, while *C.caballus* was corroborated with the original morphological description (Silva-Segundo et al. 2021). In addition, Ahern et al. (2018) reported fish eggs and larvae of *Caranxcaninus* and *C.sexfasciatus* based on molecular methods (COI), but without formal morphological analysis of specimens collected in Cabo Pulmo National Park (CPNP). The current study involved an integrative (molecular and morphological) approach to distinguishing between the several larval morphotypes of *Caranx* species.

Juveniles and adults of three nominal species of the genus *Decapterus* have been reported in the Mexican Pacific and the Gulf of California (Smith-Vaniz 1995): *Decapterusmacarellus* Cuvier, 1833; *Decapterusmacrosoma* Bleeker, 1851; and *Decapterusmuroadsi* Temminck & Schlegel, 1844. However, there is currently only a morphological description of the larval development of *D.macarellus* from specimens collected in the western central North Atlantic (Laroche et al. 2005), and *D.muroadsi* (only in postflexion stage) from specimens collected in the Colombian Pacific (Beltrán-León and Ríos-Herrera 2000). Ahern et al. (2018) identified eggs and fish larvae using molecular methods (COI) of *D.macarellus* and *D.muroadsi*. However, both species lack formal morphological description to morphologically identify the larvae. Fish larvae of the genera *Oligoplites*, *Selene*, and *Seriola* also currently lack a proper morphological description matched with DNA corroboration.

The present study uses DNA barcoding to obtain a higher resolution and precision in the identification of several fish larval morphotype sibling species of the genera *Caranx* and *Decapterus* (Family Carangidae) not adequately or not described yet. Once the taxonomic identity was confirmed molecularly, key diagnostic characters will be chosen to discriminate *Caranx* and *Decapterus* larvae at the species level from specimens collected in the southwest region of the Gulf of California. Taxonomic investigation of carangid fish larvae is a requirement for delimitating distribution patterns, spawning periods, larval drift and the species compositions of communities. All this information is helpful for the adequate management of species of commercial and ecological importance, especially in regional artisanal fisheries surrounded by the protected no-take area of Cabo Pulmo National Park.

## ﻿Material and methods

### ﻿Field work and morphological comparisons

**Figure 1.**
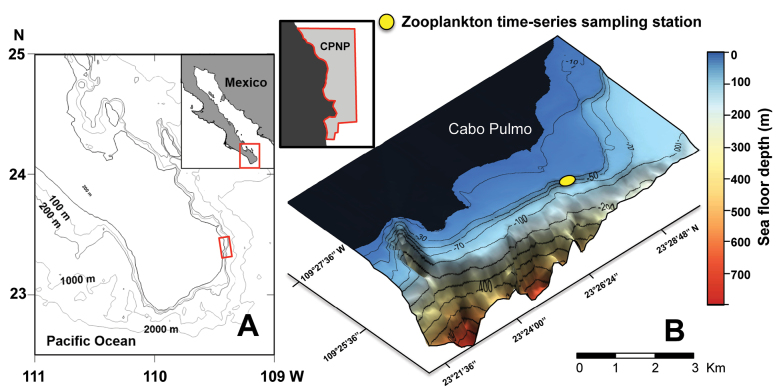
**A** Cabo Pulmo National Park (CPNP) (red outline) located in the southeast region of Baja California peninsula (inset) and **B** bathymetry of the national park measured with 120 kHz echosounder showing the locations of the weekly zooplankton time series (2016–2017; figure obtained from Ahern et al. 2018).

Analyzed fish larvae were collected from a weekly zooplankton time series conducted between January 2016 and November 2017 at Los Morros (23°27′N, 109°25′W), at Cabo Pulmo National Park (**CPNP**), the closest coral reef to the town of Cabo Pulmo (Fig. 1A, B). Zooplankton samples were collected aboard small boats, regionally known as “pangas” (5.8–8.5 m in length powered by a 200 hp outboard motor), using a standard conical net with 300 μm mesh net (0.60 m mouth diameter and 2.1 m length). The zooplankton net was towed with a rope from the “panga” stern for 10 min near the sea surface (≤ 5 m depth) with the boat angled slightly so that the tow path followed a wide arc that kept the net clear of the turbulence caused by the engine. The seafloor depth at the Los Morros site ranges between 20 and 30 m. The zooplankton net was equipped with a calibrated General Oceanics digital flowmeter (model 2030R) to estimate the filtered seawater volume to calculate the standardized abundance of fish larvae (ind. 1000 m^-3^; Smith and Richardson 1977). Zooplankton samples were passed through a 300-μm sieve on board to remove the seawater and zooplankton was preserved in 96% non-denatured ethanol, with a complete change of ethanol after 24 h for molecular analysis.

All carangid larvae were sorted from the zooplankton samples (without aliquots) and identified to the most precise taxonomic level using meristic and pigmentation characters, based on previous larval descriptions (Sumida et al. 1985; Watson et al. 1996; Beltrán-León and Ríos-Herrera 2000; Laroche et al. 2005). Several specimens were deposited in the reference collection of the Centro Interdisciplinario de Ciencias Marinas del Instituto Politécnico Nacional, La Paz, BCS, Mexico (CICIMAR-IPN). Carangid larval stages representing all collected morphotypes were selected for further DNA extraction. The total length of each larva was measured with a calibrated micrometer and photographed using a digital camera attached to a stereoscope. Digital photographs were used to draw detailed comparative schematics of the stages obtained for larvae of different total lengths.

### ﻿Molecular analysis

DNA was extracted with a modified spin-column version of the fiberglass membrane method (Ivanova et al. 2006). The fragment was amplified from the 5′ region of the mitochondrial COI gene using primers FishF2-t1 (5′-TGTAAAACGACGG CCAGTCGACTAATCATAAAGATATCGGCAC-3′) and FishR2-t1 (5′-CAGGAAACAGCTATGACACTTCAGGGTGACCGAAGAAT CAGAA-3′; Ward et al. 2005; Ivanova et al. 2007). PCR amplifications were performed in 18-μl including a 30 ng DNA template, 5× MyTaq Buffer (Bioline®), 10 μM of each primer, and 1 U of MyTaq DNA polymerase. PCR was performed in an Eppendorf Mastercycler Pro thermocycler using the following thermal cycling conditions: 3 min at 96 °C; 35 cycles of 30 s at 94 °C, 40 s at 52 °C, 1 min at 72 °C; and a final extension of 5 min at 72 °C. PCR products were visualized using electrophoresis in 2% agarose gels stained with ethidium bromide. PCR products were purified and sequenced in forward and reverse directions at the Instituto de Biología, Universidad Nacional Autónoma de México (IB-UNAM, Mexico City). All Carangidae fish larva COI sequences were manually edited and aligned using GENEIOUS® Prime 2023 software (Kearse et al. 2012).

We used the basic local alignment search tool (BLAST) included in GENEIOUS® software. COI sequences from our study were compared with COI sequences (mostly from adult specimens) previously deposited in the System of Barcode of Life Data Systems (BOLD Systems) and National Center for Biotechnology Information (NCBI) databases. We downloaded 179 COI sequences published of adult specimens of the same lengths as available nominal species of Carangidae in NCBI and BOLD Systems databases to facilitate sequence comparison (Suppl. material 1: table S1). We used DnaSP software to obtain the number of haplotypes for each species to remove redundancy in the entire sequence dataset (Rozas et al. 2003). The sequence of the common dolphinfish *Coryphaenahippurus* Linnaeus, 1758 (Coryphaenidae) downloaded from GenBank accession number MH638665 (Xu et al. 2019) was used as a cluster tree outgroup due to its close relation with the Carangidae (Smith-Vaniz 1984; Reed et al. 2002; Damerau et al. 2018). All COI sequences were aligned (ClustalW), using MEGA 10.0.5 software to calculate the intra- and inter-specific pairwise genetic distances of COI gene sequences using the Kimura 2-parameter model (K2P) and Neighbor-Joining tree reconstruction (NJ) with 10,000 bootstraps following standard methods (Kumar et al. 2016). The analysis was repeated with best-fit model and Maximum Likelihood (ML) with similar results. Any way, we decided to use the NJ and K2P methods by comparative propose with previous published articles on fish (e. g. Ward et al. 2005). Once the species were molecularly identified, the species number and abundance (ind. 1000 m^-3^) were plotted for the 2016–2017 zooplankton time series.

## ﻿Results

A total of 3,750 fish larvae were sorted out from zooplankton samples collected between 2016 and 2017. From them, 171 fish larvae (4.6%) were morphologically identified as: *Naucratesductor*, *Selarcrumenophthalmus*, *Seleneperuviana*, *Caranxsexfasciatus*, and *Caranxcaballus*, but several larvae were only identified to the genus level (*Caranx* spp., *Decapterus* spp.). For molecular analysis (COI, DNA barcoding), 57 specimens in different ontogenetic larval stages of the species identified were selected. We obtained 57 COI gene sequences of 647 bp, without stop codons, insertions, or deletions. The molecular analyses corroborated the presence of the previously identified nominal species from the morphological characteristics, but also supported the morphological distinction of three species within the genus *Caranx* (*C.caballus*, *C.caninus*, and *C.sexfasciatus*) and three distinct species of the genus *Decapterus* (*D.macarellus*, *D.macrosoma*, and *D.muroadsi*). The BLAST analysis showed eight fish larvae with high percentages of similarity with *Caranxcaballus* (99.8–100%), 18 larvae with *C.caninus* (99.7–100%), four with *C.sexfasciatus* (99.8–100%), 11 larvae with *Decapterusmacarellus* (99.7–100%), eight larvae with *D.macrosoma* (99.8–100%), one with *D.muroadsi* (99.8%), two larvae with *Naucratesductor* (98.6–100%), four larvae with *Selarcrumenophthalmus* (99.8–99.9%), and one with *Seleneperuviana* (99.6%). These 57 COI sequences were deposited in GenBank and BoldSystems (the access numbers and percentages of similarity are shown in Table 1).

**Table 1.** Molecular identification of fish larvae of the Carangidae family collected at Cabo Pulmo National Park (23°27'57.99"N, 109°24'40.99"W) during 2016–2017. BLAST results, similarity percent of COI sequences of fish larvae collected in this study with known DNA sequences databases from GenBank and BoldSystems. * = sequences previously reported in Silva-Segundo et al. (2021). BIN; barcode index number.

ID Specimen	Fish larval stages	GenBank accession number	COI Similarity (%)	Species name reported in GenBank and BoldSystems (BIN)
ILC049	preflexion	MK670988*	99.8	***Caranxcaballus*** (BOLD:AAC4853)
ILC069	preflexion	MK670991*	100	
ILC225	preflexion	MK671005*	100	
ILC233	preflexion	MT641332*	100	
ILC247	preflexion	MT641333*	100	
ILC257	preflexion	MT641334*	100	
ILC263	preflexion	MT641335*	99.8	
ILC266	flexion	MT641336*	100	
ILC051	preflexion	MK670989*	99.8	***C.caninus*** (BOLD:AAE2948)
ILC053	preflexion	MK670990*	100	
ILC111	preflexion	MK670997*	100	
ILC114	flexion	MK670998*	99.8	
ILC115	flexion	MK670999*	100	
ILC146	preflexion	MK671000*	100	
ILC219	preflexion	MK671001*	100	
ILC220	preflexion	MK671002*	100	
ILC222	preflexion	MK671003*	99.7	
ILC223	flexion	MK671004*	100	
ILC237	preflexion	MT641341*	100	
ILC238	preflexion	MT641342*	99.8	
ILC240	flexion	MT641343*	100	
ILC241	preflexion	MT641344*	100	
ILC242	preflexion	MT641345*	100	
ILC249	preflexion	MT641346*	100	
ILC254	flexion	MT641347*	99.8	
ILC262	flexion	MT641348*	99.8	
ILC226	preflexion	MT641337*	100	***C.sexfasciatus*** (BOLD:AAB0584)
ILC250	preflexion	MT641338*	99.8	
ILC251	preflexion	MT641339*	100	
ILC253	preflexion	MT641340*	99.8	
ILC047	preflexion	OR645381	99.8	***Decapterusmacarellus*** (BOLD:AAC4792)
ILC048	flexion	OR645382	100	
ILC050	preflexion	OR645383	100	
ILC054	preflexion	OR645384	99.9	
ILC221	preflexion	OR645390	100	
ILC224	preflexion	OR645391	99.8	
ILC235	flexion	OR645385	99.7	
ILC244	preflexion	OR645386	99.9	
ILC245	preflexion	OR645387	100	
ILC246	preflexion	OR645388	99.9	
ILC256	preflexion	OR645389	100	
ILC144	preflexion	OR645397	100	***D.macrosoma*** (BOLD:ADI4344)
ILC227	flexion	OR645398	100	
ILC228	preflexion	OR645399	100	
ILC248	preflexion	OR645392	99.8	
ILC252	preflexion	OR645393	99.8	
ILC261	preflexion	OR645394	99.8	
ILC264	postflexion	OR645395	99.8	
ILC265	postflexion	OR645396	100	
ILC042	preflexion	OR645400	97.2	***D.muroadsi*** (BOLD:AAC4791)
ILC110	preflexion	OR645401	100	***Naucratesductor*** (BOLD:AAE6751)
ILC112	flexion	OR645402	98.6	
ILC234	flexion	OR645404	99.9	***Selarcrumenophthalmus*** (BOLD:AAB0871)
ILC243	preflexion	OR645405	99.9	
ILC255	preflexion	OR645406	99.9	
ILC259	flexion	OR645407	99.8	
ILC113	postflexion	OR645403	99.6	***Seleneperuviana*** (BOLD:AAB7372)

**Figure 2.**
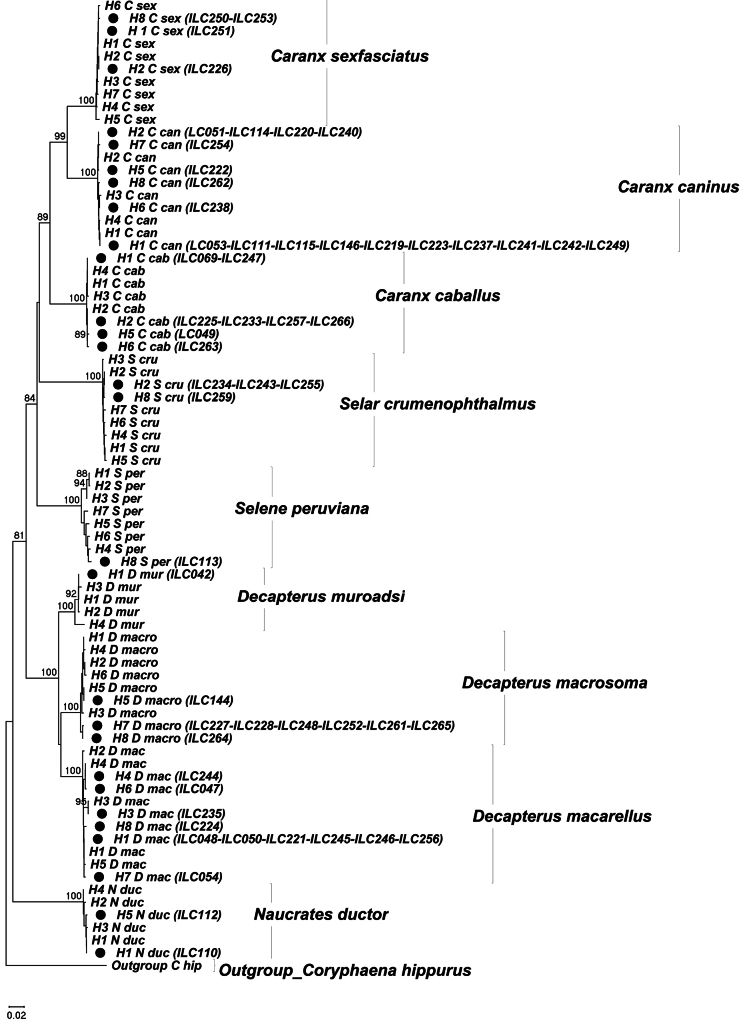
Neighbor-Joining tree of the family Carangidae using the Kimura 2-parameter model based on the haplotypes of Cytochrome C oxidase subunit 1 (COI), see Suppl. material 1: table S1. Black dot: Haplotypes of the carangid larvae from the present study. Acronyms: *C sex* (*Caranxsexfasciatus*); *C can* (*Caranxcaninus*); *C cab* (*Caranxcaballus*); *S cru* (*Selarcrumenophthalmus*); *S per* (*Seleneperuviana*); *D mur* (*Decapterusmuroadsi*); *D macro* (*Decapterusmacrosoma*); *D mac* (*Decapterusmacarellus*); *N duc* (*Naucratesductor*); and *C hip* (*Coryphaenahippurus*).

A total of 57 COI sequences from the present study and 179 sequences downloaded from GenBank and BoldSystems were aligned. From this database, the representative haplotypes for each species were obtained (Suppl. material 1: table S1). These haplotypes were used for the analysis of genetic distances and the reconstruction of the Neighbor-Joining tree (NJ). Intraspecific genetic distances (K2P model) ranged between 0.20% and 1.34% and interspecific differences of species of the same genus varied between 8.4% and 11.7% among *Caranx* and between 6.4% and 6.5% among *Decapterus* species. The distances between genera showed values between 14.2% and 22.8% (Suppl. material 1: table S2). The reconstruction of the NJ tree showed five main clusters corresponding to the five genera found in the present study (*Caranx*, *Decapterus*, *Naucrates*, *Selar*, and *Selene*). The clusters of the genera *Caranx and Decapterus* showed three subgroups corresponding to their respective species (*C.caballus*, *C.caninus*, and C. *sexfasciatus*; *D.macarellus*, *D.macrosoma*, and *D.muroadsi*; Fig. 2). All the COI sequences of the present study were grouped in their representative clusters (comparing with adults of each species), corroborating the specific identification of the larvae collected in CPNP during 2016–2017 (Fig. 2). The morphological characteristics of *Naucratesductor*, *Selarcrumenophthalmus*, and *Seleneperuviana* larvae identified in the present work were not described here because complete morphological descriptions of their larval development are already available in Watson et al. (1996). However, it was necessary to provide detailed descriptions of pigmentation patterns and diagnostic characters of fish larvae of the genera *Caranx* and *Decapterus* because there is little information on the first larval stages of these two genera. Here we describe the diagnostic characters that allow species distinction among *Caranx* larvae (*Caranxcaballus*, *C.caninus*, and *C.sexfasciatus*) and among *Decapterus* larvae (*Decapterusmacarellus*, *D.macrosoma*, and *D.muroadsi*). When it was not possible to count fin rays and spines, information from the taxonomic guides for each species was used.

### ﻿Diagnostic characters of larvae of Carangidae

#### ﻿Genus Caranx

Taxon classificationAnimaliaPerciformesCarangidae

Lacepède, 1801

D8D1D780-9C9D-5BD4-A06F-224166D311C1

Caranx caballus Günther, 1868

##### Material examined.

Eight larvae identified as *C.caballus*. Seven in preflexion stage (2.0–3.2 mm) and one in flexion (3.7 mm). Myomeres 25 (10 precaudal and 15 caudal). Dorsal and anal fin not developed in preflexion and flexion stages. Meristic data for *Caranxcaballus* larvae reported by Watson et al. (1996): 10 precaudal vertebrae and 15 caudal (25 total); number of fin spines and rays, dorsal VIII + I, 21–25, and anal II + I, 17–24.

##### Preflexion larvae 2.5 mm total length.

**Figure 3.**
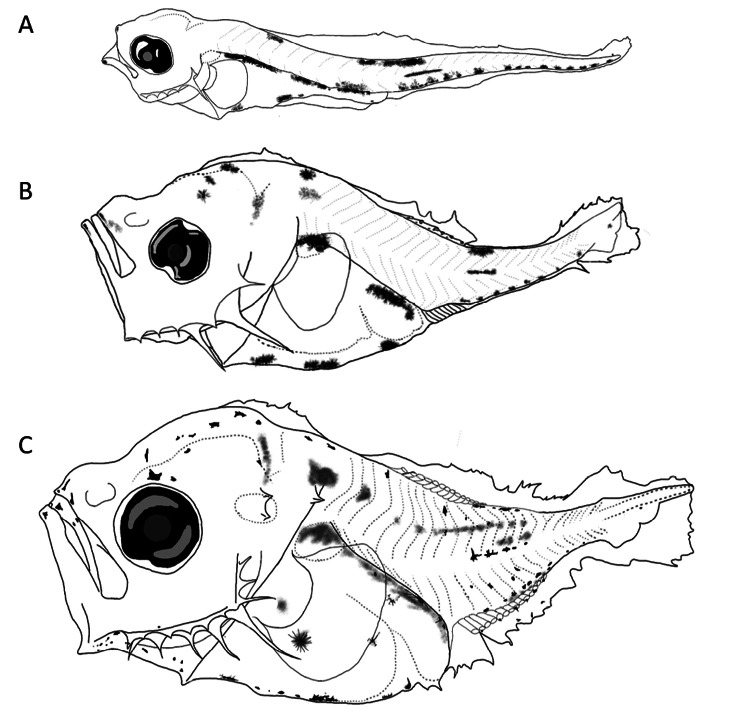
First larval stages of *Caranxcaballus***A** preflexion, 2.5 mm **B** preflexion, 3.2 mm **C** flexion, 3.7 mm total length.

**(Fig. 3A)** Body slender, second spine at preopercular angle large. ***Pigmentation***: lower jaw with pigment; upper edge of gut; small dorsal pigment between third and fifth myomeres, large pigments between 9–12 myomeres; in lateral midline between myomeres 12–15; ventrally, one posterior to cleithral symphysis, central portion of gut, one close to anus, and postanal series from the second postanal myomere to notochord. Pattern of pigments present in larvae <3.7 mm.

##### Preflexion larvae 3.2 mm.

**(Fig. 3B)** Deeper body with broad crest from parietal to supraoccipital region, which is not high. Two robust preopercular spines at preopercular angle. ***Pigmentation***: Three pigments on parietal and supraoccipital region; internal pigment on brain and upper margin of gut (swim bladder and terminal section).

##### Flexion larvae 3.7 mm.

**(Fig. 3C)** Slightly higher supraoccipital crest and one pair of pterotic and supracleithral spines. Preopercular spines long and robust at the base; thin at ends. ***Pigmentation***: Pigments scattered over lower jaw tip and chin; over head, from eye (upper edge) to supraoccipital region; ventrally, between lower jaw angle and cleithra; upper margin of gut completely pigmented, like ventral margin and central part (lateral view); on trunk, dispersed from dorsal pteryogyophores to lateral midline, with internal pigments between lateral midline and dorsum. Pattern consistently matches original larvae of *Caranxcaballus* (Sumida et al. 1985).

##### Main characters.

Dorsal pigment located between myomeres 3–5, strong pigmentation between myomeres 9–12; in lateral midline between myomeres 12–15 and three ventral pigments (posterior to cleithral symphysis, central portion of gut, and close to anus) followed by postanal series from 2^nd^ postanal myomere to notochord.

#### ﻿ Caranx caninus

Taxon classificationAnimaliaPerciformesCarangidae

Günther, 1867

76B4402E-BA8E-53D8-8F48-E643BFC0C2D3

##### Material examined.

Eighteen specimens of *C.caninus*, 12 in preflexion and 6 in flexion, from 2.5 mm to 4.1 mm total length. Myomeres 24 (10 precaudal and 14 caudal). Fin elements not developed. Meristic data for *C.caninus* larvae reported in Watson et al. (1996): 10 precaudal vertebrae and 14 caudal (24 total); number of fin spines and rays, dorsal VII-VIII + I, 18–23, and anal II + I, 18–21.

##### Preflexion larvae 2.5 mm total length.

**Figure 4.**
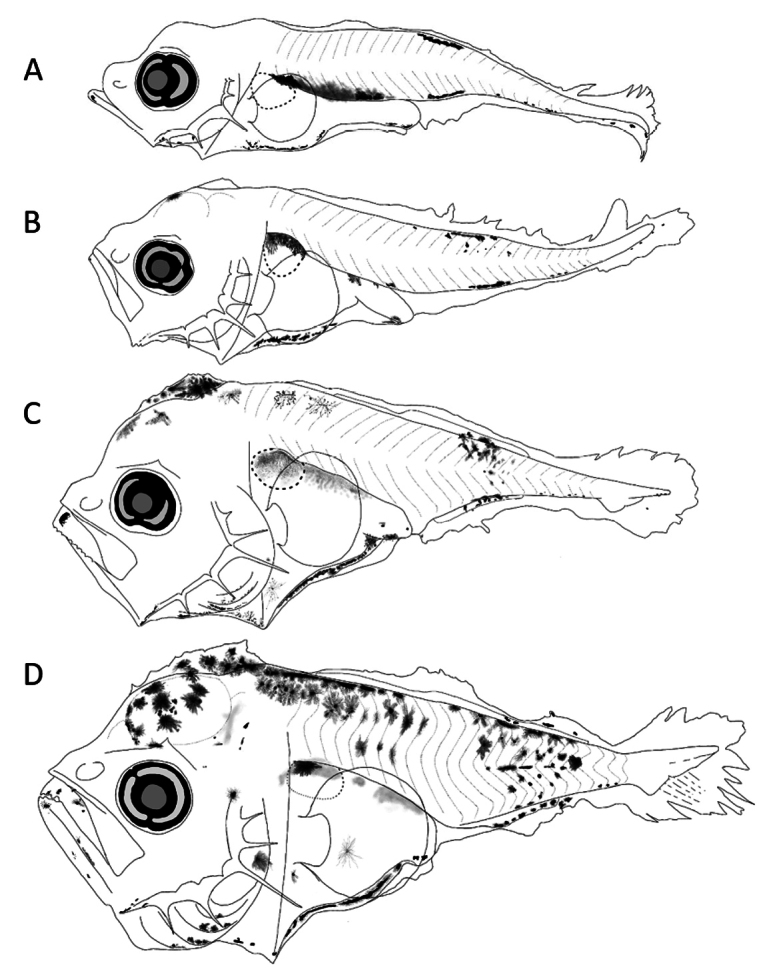
First larval stages of *Caranxcaninus***A** preflexion, 2.5 mm **B** preflexion, 3.2 mm **C** preflexion, 3.4 mm **D** flexion, 4.1 mm total length.

**(Fig. 4A)** Small supraocular crest, five spines on preopercular edge (third and fourth longer and more robust than others). ***Pigmentation***: Pigments on tip of lower jaw; mandibular angle and branchiostegal membrane; intensified on upper margin of gut over swim bladder, several on ventral margin of gut coil, one on terminal section anterior to anus, heavier and extended in larger sizes. Dorsal and ventral pigment series between myomeres 12–15, followed by small pigments until notochordal tip, three of which appear on lower lobe of caudal fin primordium.

##### Preflexion larvae 3.2 mm.

**(Fig. 4B)** Head slightly deeper, with supraoccipital crest short and slightly raised base, preopercular spines more elongated and supraocular ridge with crescent tip. ***Pigmentation***: Pigments above head, on midbrain; dorsal and ventral dispersed between myomeres 12–17; on upper edge of gut sparse, but heavy on dorsal swim bladder.

##### Preflexion larvae 3.4 mm.

**(Fig. 4C)** Head and trunk deeper, supraocular ridge larger with pronounced tip, preopercular spines thin, elongated and more pronounced in angle. ***Pigmentation***: Pigments increasing on head compared to smaller sizes; supraoccipital crest pigmented; on chin; in branchiostegal membrane and rays; on upper edge of gut disperse, but heavy and intense on lower margin, marked at level of anus; on trunk (dorsally); above lateral midline (myomeres 3–5), and another series in myomeres 13–18 strongly pigmented and widespread from dorsal to anal pterygiophores; and ventral series of small pigments in caudal region, one on lower lobe of caudal fin fold, and three at lower margin of notochordal tip.

##### Flexion larvae 4.1 mm.

**(Fig. 4D)** Deeper body compared with smaller sizes, with supraoccipital crest extended between frontal to occipital area, with serrated edge. Supraocular crest comparatively high tip, and preopercular spines thinner and elongated, mainly in the preopercular angle. ***Pigmentation***: More pigments in jaws; on branchiostegal rays; over brain and crest; in upper and ventral gut margin, and in most of trunk (including some on lateral midline); except in central (myomeres 9–12) and caudal region without pigmentation.

##### Main characters.

Pigmentation on dorsal and ventral series between myomeres 12–15; gut, several on ventral margin of gut coil, one on terminal gut section anterior to anus; and pigmented supraoccipital crest in larvae > 3.4 mm.

#### ﻿ Caranx sexfasciatus

Taxon classificationAnimaliaPerciformesCarangidae

Quoy & Gaimard, 1825

52A3EC65-45EF-569D-BA2C-08C067AE84BA

##### Material examined.

Four larvae in preflexion stages identified as *C.sexfasciatus* ranging from 2.8 to 4 mm. Myomeres 25 (10 precaudal and 15 caudal), fin elements not possible to quantify (spines or rays). *C.sexfasciatus* reported by Watson et al. (1996): 10 precaudal vertebra and 15 caudal (25 total); number of fin spines and rays, dorsal VII-VIII + I, 19–22, and anal II + I, 14–17.

##### Preflexion larvae 2.8 mm total length.

**Figure 5.**
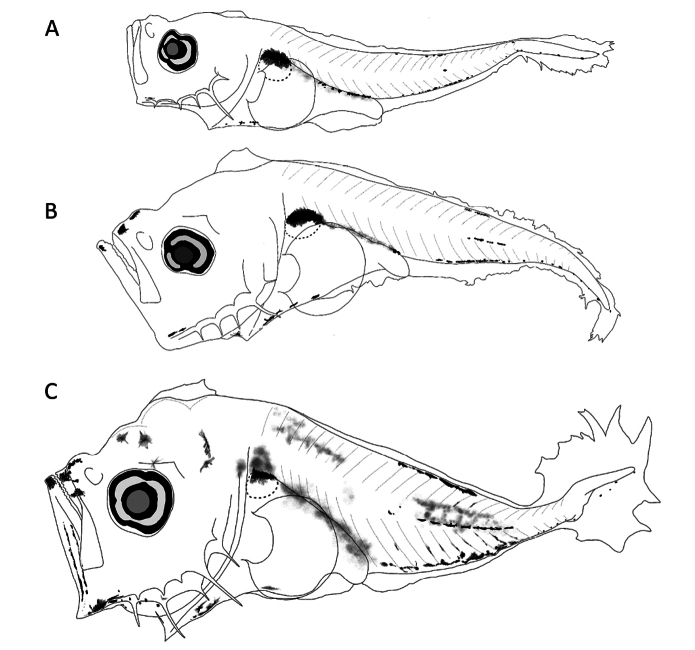
First larval stages of *Caranxsexfasciatus***A** preflexion, 2.8 mm **B** preflexion, 3.2 mm **C** preflexion, 3.5 mm total length.

**(Fig. 5A)** Slender trunk, slightly deep head, supraoccipital crest with short base and without pigmentation, well-developed preopercular edge spines, mainly angle spine. ***Pigmentation***: Scarce on almost entire body; present in mandibular angle; internal in upper margin of gut and some on swim bladder; no pigmentation ventrally; and few posteriors to cleithral symphysis. On trunk, several on dorsal edge between 11–17 myomeres and small pigments in lateral midline; and postanal to end of notochord.

##### Preflexion larvae 3.2 mm.

**(Fig. 5B)** Deepest head, supraoccipital crest slightly higher, supraocular spine with pointed elevation. ***Pigmentation***: On snout tip; mandibular angle; anterior to cleithral symphysis; ventrally over middle of gut; and lateral midline.

##### Preflexion larvae 3.5 mm.

**(Fig. 5C)** Deeper head and trunk, with broad-based supraoccipital crest; and longer and thinner spines in preopercular margin. ***Pigmentation***: Denser than in smaller sizes; supraoccipital crest not pigmented; on head, at forebrain and nape; marked in tip of jaws; over lower angle of jaw; and lower margin of preopercular spines and branchiostegal membrane; on trunk, internally over first seven myomeres and lateral midline.

##### Main characters.

Less pigmented larvae compared with *C.caninus* and *C.caballus*. Dorsal series of pigments between 11–17 myomeres and in lateral midline. Scarce ventral pigmentation on gut and no pigmentation on supraoccipital crest, in all sizes.

#### ﻿Genus Decapterus

Taxon classificationAnimaliaPerciformesCarangidae

Bleeker, 1851

6EBA2787-1E57-56D0-8249-FF95EC0E0F76

Decapterus macarellus Cuvier, 1833

##### Material examined.

Eleven larvae identified as *D.macarellus*, 9 in preflexion (2.2–3.5 mm) and 2 in flexion (4.2–5.0 mm). Myomeres 24 (10 precaudal and 14 caudal). Dorsal and anal fin not developed. *D.macarellus* larval meristic data reported in Watson et al. (1996): 10 precaudal vertebrae and 14 caudal; number of fin spines and rays, dorsal VIII + I, 30–36+1, and anal II + I, 26–30+1.

##### Preflexion larvae 2.2 mm total length.

**Figure 6.**
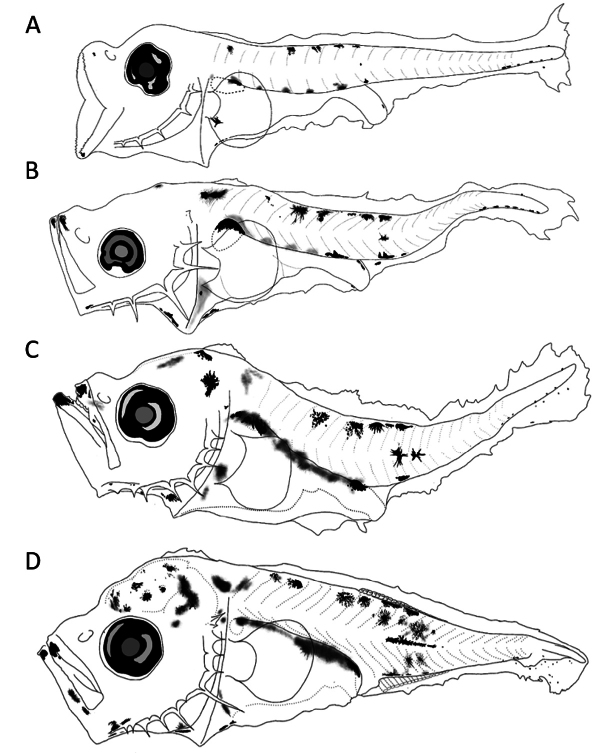
First larval stages of *Decapterusmacarellus***A** preflexion, 2.2 mm **B** preflexion, 2.7 mm **C** preflexion, 3.5 mm **D** flexion, 4.2 mm total length.

**(Fig. 6A)** Slender and elongated body, with short and thinner preopercular spines. ***Pigmentation***: On jaws tip; cleithral symphysis; below pectoral fin; on trunk, parallel series on dorsal and upper gut margin; on lateral midline a single pigment; on caudal zone, a series from last two myomeres to end of notochord, in all forms; and one above notochord tip.

##### Preflexion larvae 2.7–3.5 mm.

**(Fig. 6B, C)** Elongated and deeper body. ***Pigmentation***: Pattern like the smallest (2.2 mm), unlike one pigment in mandibular angle, on head, in section anterior and posterior to cleithral symphysis, and lateral midline intensifies.

##### Flexion larvae 4.2 mm.

**(Fig. 6D)** Noticeably deeper body, with thin spines on preopercular edge, and one elongated at angle; with broad supraoccipital crest at its base, with low height and irregular serrated profile. ***Pigmentation***: Denser on head; some on lower jaw profile; a series marked in dorsal margin; streaks on dorsum and ventrum extended on each side of dorsal an anal fins base; and scattered in the lower lobe of caudal finfold.

##### Main characters.

On trunk, parallel series of pigments marked on dorsal profile and on upper gut margin; and one pigment at lateral midline in small sizes; increased pigmentation with age.

#### ﻿ Decapterus macrosoma

Taxon classificationAnimaliaPerciformesCarangidae

Bleeker, 1851

E48E7A3D-38E7-53FD-AA65-BEBCBFDA2036

##### Material examined.

Eight larvae identified as *D.macrosoma*, 5 in preflexion, one in flexion, and 2 in postflexion stages (2.0–8.1 mm). Myomeres 24 (10 precaudal and 14 caudal). Fins not fully developed, but it was possible to count: VII + I, 29 elements in dorsal fin and II + 26 in anal fin. Meristic information reported in Watson et al. (1996): number of fin spines and rays, dorsal VIII + I, 32–38+1, and anal II + I, 26–30+1; and 10 precaudal and 14 caudal vertebrae.

##### Preflexion larvae 2–2.9 mm total length.

**Figure 7.**
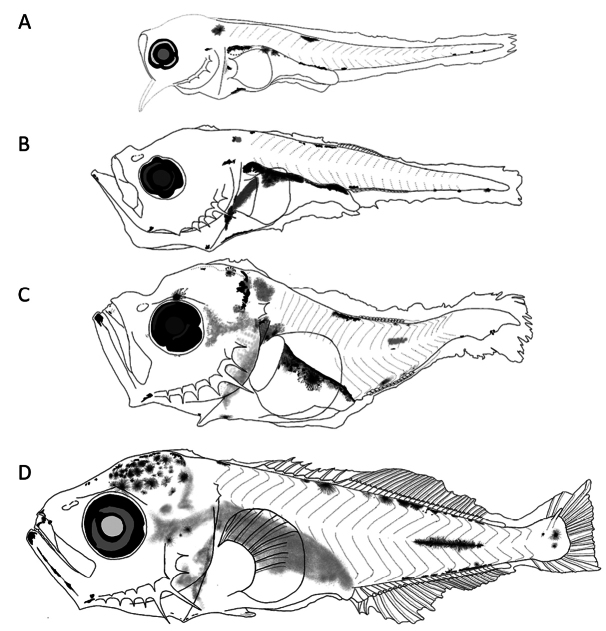
First larval stages of *Decapterusmacrosoma***A** preflexion, 2 mm **B** preflexion, 2.9 mm **C** flexion, 3.8 mm **D** postflexion, 8.1 mm total length.

**(Fig. 7A, B)** Slender and elongated bodies, slightly more robust in 2.9 mm; supracleithral crests with short base and lower height; with short preopercular spines and elongated one at angle. ***Pigmentation***: On head just behind nape; chin; mandibular angle; above cleithral symphysis; at upper margin of opercular area; on gut and upper margin mainly over swim bladder and anterior margin of gut; on trunk, on upper margin between myomeres 7–9; postanal, just posterior to anus; three more on ventral margin of notochord tip.

##### Flexion larvae 3.8 mm.

**(Fig. 7C)** Slightly increase in body depth. Supraoccipital ridge base comparatively broad with slightly serrated margin, one pair of supracleithral spines, comparatively shorter preopercular spines than other species, robust base in angle. ***Pigmentation***: On head, stronger over forebrain and nape and internally-placed posterior to eye; in lateral midline internal and contracted; on trunk, over dorsal and ventral margins; one at center of hypural plate.

##### Postflexion larvae 8.1 mm.

**(Fig. 7D)** Elongated and slightly deep bodies. Supraoccipital and supraocular crests and supracleithral spines reduced, preopercular spines larger and more robust. ***Pigmentation***: concentrated on head, internal pigments behind eye; on jaws tips and its lower margin; up to mandibular angle and around cleithral symphysis; on anterior and upper margin of gut; on trunk, dorsal series extended to myomere 20; over lateral midline and anal fin base; three stellate melanophores in hypural plate.

##### Main characters.

Pigmentation behind the nape; on upper margin between myomeres 7–9; ventrally, one postanal just before anus and three more on notochord tip (< 3.8 mm). In postflexion stages, it extends in dorsal and ventrally margins, lateral line, and three stellate pigments in center of hypural plate.

#### ﻿ Decapterus muroadsi

Taxon classificationAnimaliaPerciformesCarangidae

Temminck & Schlegel, 1844

868C3866-941C-5E19-8F16-7ED3B7711D5F

##### Material examined.

One larva in preflexion identified as *D.muroadsi*. Myomeres 24 (10 precaudal and 14 caudal). Fins development not observed. Meristic data for *D.muroadsi* larvae have been reported by Watson et al. (1996): 10 precaudal vertebrae and 14 caudal (24 total); number of fin spines and rays, dorsal VII-VIII + I, 29–33+1, and anal II + I, 25–29+1.

##### Preflexion larvae 3.2 mm total length.

**Figure 8.**
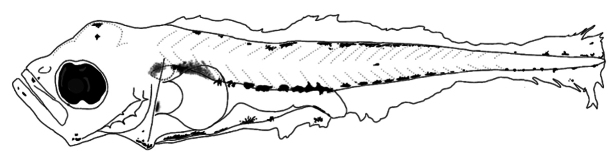
First larval stages identified of *Decapterusmuroadsi*: preflexion, 3.2 mm total length.

**(Fig. 8)** Elongated body, with short preopercular spines. ***Pigmentation***: Heavier on dorsal and ventral margin than other species of *Decapterus*; on head; in both jaws tips, mandibular angle and cleithral symphysis; upper margin of gut which merges with those of trunk; over gas bladder; and ventrally on gut and finfold near to anus; on trunk, in lateral midline (myomere 14); parallel from dorsal and ventrally profile; and around lower and upper lobes of caudal finfold.

##### Main characters.

Preflexion larvae more pigmented than other *Decapterus* species, with line of pigments on dorsal and ventral margins along almost entire contour, ventrally pigmented in gut, and one anterior to anus over finfold.

### ﻿Species abundance during 2016–2017 weekly time series

**Figure 9.**
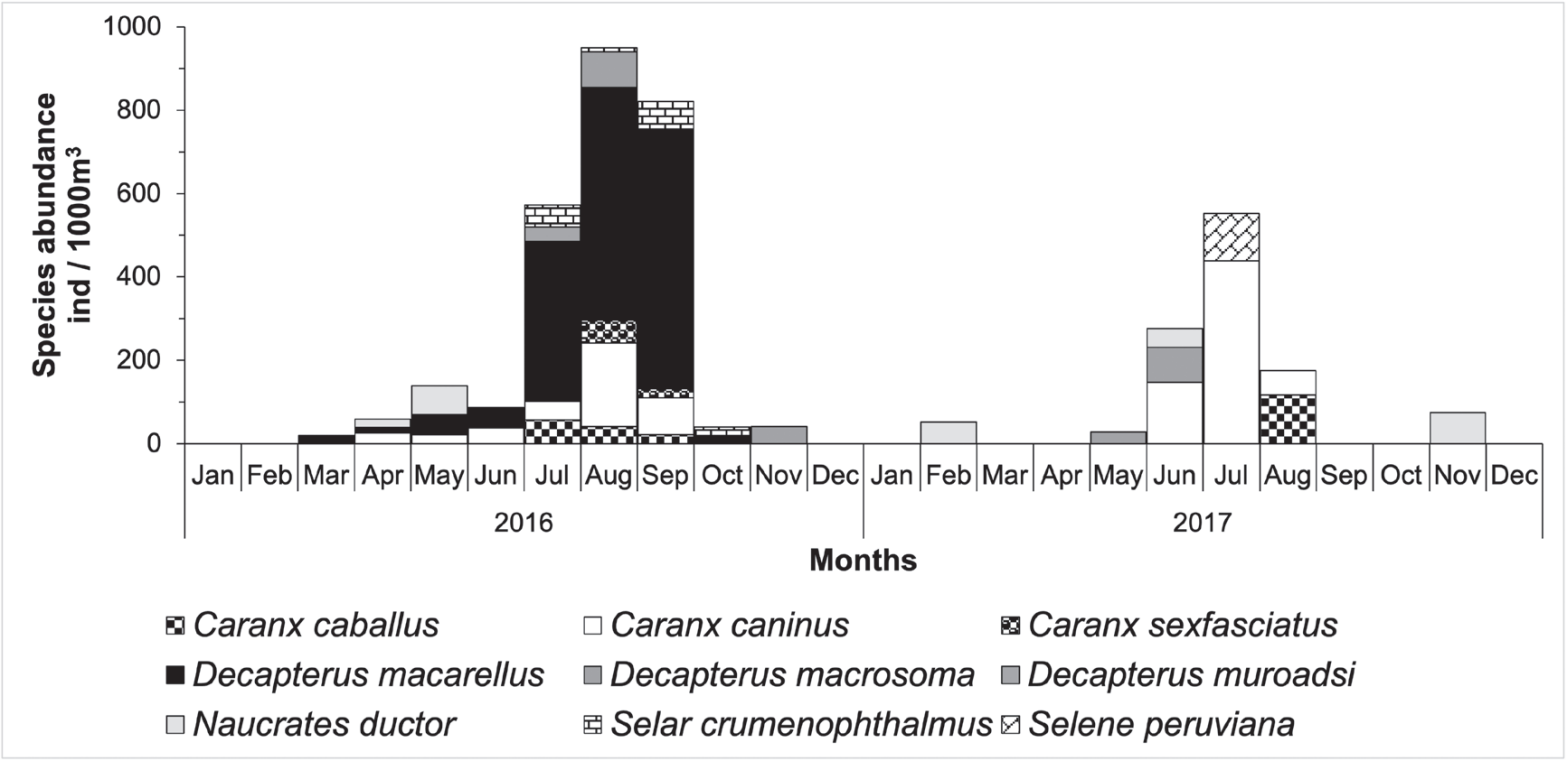
Larval abundance of the family Carangidae from Cabo Pulmo National Park, Mexico, surveyed weekly during 2016–2017.

Larval Carangidae were collected during most of 2016, except in winter (December–February; Fig. 9). All species, except *S.peruviana*, were observed during 2016. The highest species number and abundance were observed during summer 2016 (July–September) with *Decapterusmacarellus* being the most abundant in September (622 ind. 1000m^-3^). During 2017, species number and abundance decreased and were recorded mostly during June–August (*Caranxcaballus*, *C.caninus*, *D.macrosoma*, *Naucratesductor* and *Seleneperuviana*). *Caranxcaninus* was the most abundant species (438 ind. 1000 m^-3^ in July). *Naucratesductor* and *D.macrosoma* are newly recorded species for the CPNP region, not previously reported in checklists in any life phase (egg, larvae, adult). The presence of larval stages of *N.ductor* and *D.macrosoma* larvae during both years suggest that both species are residents of the CPNP (Fig. 9).

## ﻿Discussion

The present study provides the first morphological and molecular evidence to distinguish three sibling species of the genus *Caranx*, complementing molecular descriptions previously reported in Silva-Segundo et al. (2021). In addition, diagnostic characters to distinguish larvae of three species of *Decapterus* not previously described are provided. These descriptions rapidly and accurately delimit six fish larvae species without the need for molecular methods, facilitating future ecological research.

The juvenile and adult ichthyofauna at Cabo Pulmo National Park has been monitored since it was founded in 1995 as a no-take protected national park (Reyes-Bonilla 1997; Aburto-Oropeza et al. 2011, 2015; Olivier et al. 2022). Currently, the reef ecosystem is healthy, with notable increases in the diversity, abundance, and biomass of fish species relative to 1995 (Alvarez-Filip et al. 2006; Alvarez-Filip and Reyes-Bonilla 2006; Saldívar-Lucio and Reyes-Bonilla 2011; Aburto-Oropeza et al. 2011, 2015; Ahern et al. 2018; Olivier et al. 2022). The fish taxonomic checklists of CPNP obtained from visual censuses of adult fish showed the presence of 14 species of carangids (Villarreal-Cavazos et al. 2000; Ayala-Bocos et al. 2018). Subsequently, based on molecular data from eggs and larvae, three more species of Carangidae were recorded in the CPNP (*Ferdauiaorthogrammus*, *Decapterusmuroadsi*, and *Seleneperuviana*; Ahern et al. 2018). In the present study, *D.macrosoma* and *Naucratesductor* are added to the checklist, recording a total of 19 species of Carangidae at the CPNP (Suppl. material 1: table S3).

Adults of five species of the *Caranx* genus have been recorded in the Mexican Pacific region: *Caranxcaninus*, *C.caballus*, *C.melampygus*, *C.lugubris*, and *C.sexfasciatus* (Smith-Vaniz 1995; Allen and Robertson 1998; Froese and Pauly 2000). The larvae of *C.caballus* and *C.sexfasciatus* are the only species with larval descriptions (Sumida et al. 1985; Beltrán-León and Ríos-Herrera 2000). The larvae of *C.caballus* presented a pigmentation pattern and meristic elements like the original description by Sumida et al. (1985). However, in the present study, the larvae also have a small series of dorsal pigmentation that extends between the third and fifth myomeres in all sizes.

The larvae of *Caranxsexfasciatus* identified in Sumida et al. (1985), were larvae of *C.caninus* based on molecular evidence reported in Silva-Segundo et al. (2021), the latter species without formal description until the present study. The integration of morphological data and DNA barcoding of *C.caninus* and *C.sexfasciatus* has allowed us to detect the main diagnostic features separating both species larvae. The larvae of *C.caninus* have characteristic melanophores along the ventral margin of the gut, dorsal and ventral series of melanophores between myomeres 12–15 forming a vertical stripe-like which expand according to larval development, with melanophores on the head and supraoccipital crest since larvae of 3.4 mm. In contrast, the larvae of *C.sexfasciatus* showed little pigmentation in the cleithral symphysis region, few and small melanophores in the dorsal and ventral part, with a small pigment in the midline, in the middle of the body, which spreads as the larvae grow. In addition, the larvae have few pigments on the dorsal margin and some postanal pigments distributed to the end of the notochord.

Adults of *Decapterusmacarellus*, *D.macrosoma*, and *D.muroadsi* were previously recorded in the Mexican Pacific (Smith-Vaniz 1995; Allen and Robertson 1998; Froese and Pauly 2000). According to Watson et al. (1996), *Decapterus* sp. could correspond to *D.macrosoma* based on the meristic information and pigmentation pattern, which was corroborated with larvae collected in the present study based on their high genetic similarity with sequences of adults of *D.macrosoma*. Although the larvae of *D.macarellus* had not been recorded in the Mexican Pacific, sequenced specimens of the species showed a similar pattern of pigmentation with larvae previously described by Laroche et al. (2005) in the Atlantic, confirming its circumglobal tropical distribution pattern. The preflexion larvae identified in the present study as *D.muroadsi* using DNA barcoding showed a pigmentation pattern considerably distinct from the larvae of *D.macarellus* and *D.macrosoma*, with melanophores along almost the entire dorsal and ventral profile of the trunk (Laroche et al. 2005). Our *D.muroadsi* larvae was like those reported by Steinke et al. (2016) from South Africa. Additionally, a similar pattern of pigmentation was observed in another postflexion larva (8.8 mm) collected in the Colombian Pacific (Beltrán-León and Ríos-Herrera et al. 2000).

The present study provides larval descriptions in different ontogenetic stages of comparative development among three species of *Caranx* (*C.caballus*, *C.caninus*, and *C.sexfasciatus*). However, it is necessary to mention that the larval stages of two other species present in the southern region of the Gulf of California (*C.melampygus* and *C.lugubris*) are still unknown and need to be morphologically and molecularly identified. Therefore, it is necessary to continue analyzing zooplankton samples and increase the taxonomic information of the group of carangid larvae. The combination of morphological and molecular taxonomic methods allowed us to find and distinguish pigmentation patterns that can be used as diagnostic features to separate commercially important fish species (*Caranx* and *Decapterus*). In addition, more precise information about species number and abundance of the larvae of carangid species from the Cabo Pulmo National Park is now available, which can be used for future management and conservation plans of these species that are an artisanal fishing resource outside the national park.

## Supplementary Material

XML Treatment for Caranx

XML Treatment for Caranx caninus

XML Treatment for Caranx sexfasciatus

XML Treatment for Decapterus

XML Treatment for Decapterus macrosoma

XML Treatment for Decapterus muroadsi

## ﻿Additional information

### Author ORCIDs

Claudia A. Silva-Segundo https://orcid.org/0000-0002-5938-0576

René Funes-Rodríguez https://orcid.org/0000-0002-6139-0861

Eduardo Anaya-Godínez https://orcid.org/0000-0002-8228-5905

Jaime Gómez-Gutiérrez https://orcid.org/0000-0003-2516-897X

### Data availability

All of the data that support the findings of this study are available in the main text or Supplementary Information. Data will be available upon a reasonable request to corresponding author. Biological material was deposited on the ichthyoplankton collection of the CICIMAR-IPN and COI sequences are deposited in NCBI and Bold System (accession numbers see Table 1).

## Supplementary materials

Supplementary material 1 Supplementary data Data type doc Explanation note Table of haplotype, genetic distance and references. https://binary.pensoft.net/file/1134978This dataset is made available under the Open Database License (http://opendatacommons.org/licenses/odbl/1.0/). The Open Database License (ODbL) is a license agreement intended to allow users to freely share, modify, and use this Dataset while maintaining this same freedom for others, provided that the original source and author(s) are credited.Claudia A. Silva-Segundo, René Funes-Rodríguez, Eduardo Anaya-Godínez, Jaime Gómez-Gutiérrez
