# Supplementary material for: ﻿Molecular and morphological identification of larvae of Carangidae (Teleostei, Carangiformes) species from southern Gulf of California
Source: Zookeys. 2024 Sep 16;1212:195–215. doi: 10.3897/zookeys.1212.118644 (PMC11420543; doi:10.3897/zookeys.1212.118644)
Supplement: Supplementary material 1 — Supplementary data [file zookeys-1212-195_article-118644__-s001.doc]

**Supplementary material**

**Table S1.** Haplotypes of mitochondrial gene cytochrome c oxidase subunit I (COI) of species of the Carangidae family obtained from sequences from present study (*, additionally shown in bold font) and reference sequences retrieved from GenBank and BoldSystems. All haplotypes were compared for intra- and inter-specific genetic distances analysis and Neighbor-Joining tree reconstruction. References: ^1^ International Barcode of Life (IBOL/BOLSYSTEMS), ^2^ (Yancy et al. 2008), ^3^ (Hubert et al. 2012), ^4^ (Steinke et al. 2016), ^5^ (Hou et al. 2018), ^6^ (Chang et al. 2017), ^7^ (Ward and Holmes 2007), ^8^ (Mat Jaafar et al. 2012), ^9^ (Zhang and Hanner 2011), ^10^ (Templonuevo et al. 2018), ^11^ (Dahruddin et al. 2017), ^12^ (Xu et al. 2019), ^13^ (Kimura et al. 2022), ^14^ (Jaonalison et al. 2022), ^15^ (Silva-Segundo et al. 2021), ^16^ (Limmon et al. 2020), ^17^ (Andriyono et al. 2020), ^18^ (Asgharian et al. 2011), ^19^ (Hou et al. 2021), ^20^ (Hastings and Burton 2008), ^21^ (Robertson et al. 2017), ^22^ (Hou et al. 2020), ^23^ (Huang et al. 2022), ^24^ (Lakra et al. 2011), ^25^ (Mat Jaafar et al. 2020), ^26^ (Rathnasuriya et al. 2021). Acronyms: *C sex (Caranx sexfasciatus); C can (Caranx caninus); C cab (Caranx caballus); S cru (Selar crumenophthalmus); S per (Selene peruviana); D mur (Decapterus muroadsi); D macro (Decapterus macrosoma); D mac (Decapterus macarellus); N duc (Naucrates ductor)*; and *C hip* (*Coryphaena hippurus*).

| HAPLOTYPE | # sec | GenBank Accession Number |
| --- | --- | --- |
| ***Caranx caballus*** | | |
| *H1 C cab* | 9 | (RDFCA268-05, RDFCA270-05, RDFCA272-05, RDFCA384-05)^1^; (LC646707)^13^; (MK670995, MK670996)^15^ |
|  | ***2** | **(MK670991-ILC069, MT641333-ILC247)^15^** |
| *H2 C cab* | 1 | (RDFCA393-05)^1^ |
|  | ***4** | **(MK671005-ILC225, MT641332-ILC233, MT641334-ILC257, MT641336-ILC266)^15^** |
| *H3 C cab* | 1 | (RDFCA269-05)^1^ |
| *H4 C cab* | 1 | (RDFCA271-05)^1^ |
| *H5 C cab* | ***1** | **(MK670988-ILC049)^15^** |
| *H6 C Cab* | ***1** | **(MT641335-ILC263)^15^** |
| ***Caranx caninus*** | | |
| *H1 C can* | 6 | (RDFCA324-05, RDFCA392-05, HQ974525, HQ974567)^1^; (EU752066)^2^; (MK670993)**^15^** |
|  | ***10** | **(MK670990-ILC053, MK670997-ILC111), MK670999-ILC115, MK671000-ILC146, MK671001-ILC219, MK671004-ILC223, MT641341-ILC237, MT641344-ILC241, MT641345-ILC242, MT641346-ILC249)^15^** |
| *H2 C can* | 4 | (JN313923, RDFCA231-05, RDFCA385-05)^1^; (MK670994)^15^, |
|  | ***4** | **(MK670989-ILC051, MK670998-ILC114, MK671002-ILC220, MT641343-ILC240)^15^** |
| *H3 C can* | 1 | (EU752067)^2^ |
| *H4 C can* | 1 | (MK670992)^15^ |
| *H5 C can* | ***1** | **(MK671003-ILC222)^15^** |
| *H6 C can* | ***1** | **(MT641342-ILC238)^15^** |
| *H7 C can* | ***1** | **(MT641347-ILC254)^15^** |
| *H8 C can* | ***1** | **(MT641348-ILC262)^15^** |
| ***Caranx sexfasciatus*** | | |
| *H1 C sex* | 13 | (OL409659)^14^; (MN870438)^16^; (MH085891)^17^; (MH638724)^12^; (KU692409)^11^; (KU176404)^4^; (JX261315, JX261259, HQ560966, HQ560947)^8^; (JQ431548)^3^; (JN312936)^1^; (EF609305)^7^ |
|  | ***1** | **(MT641339-ILC251)^15^** |
| *H2 C sex* | 10 | (MN869936)^16^; (LC646717)^13^; (KU692408)^11^; (JX261569, JX261414, HQ560961)^8^; (JQ431547)^3^; (JN312937)^1^; (JF952695)^9^; (HQ149821)^18^ |
|  | ***1** | **(MT641337-ILC226)^15^** |
| *H3 C sex* | 2 | (MH085890)^17^; (KU176334)^4^ |
| *H4 C sex* | 1 | (OL409240)^14^ |
| *H5 C sex* | 1 | (MN870366)^16^ |
| *H6 C sex* | 1 | (MN870120)^16^ |
| *H7 C sex* | 1 | (KC970458)^10^ |
| *H8 C sex* | ***2** | **(MT641340-ILC253, MT641338-ILC250)^15^** |
| ***Decapterus macarellus*** | | |
| *H1 D mac* | 22 | (KU943796, KU943797, KU943798)^6^; (MH085883, MH085884)^17^; (MH638676, MH638686, MH638719, MH638731, MH638732, MH638733, MH638755, MH638772, MH638781)^12^; (MT609955, MT609956)^22^; (MW379446)^19^; (OL512829, OL512839, OL512842, OL512854, OL512881)^23^ |
|  | ***6** | **ILC048, ILC050, ILC221, ILC245, ILC246, ILC256** |
| *H2 D mac* | 2 | (MH638698)^12^; (MW379539)^19^ |
| *H3 D mac* | 1 | (MH085882)^17^ |
|  | ***1** | **ILC235** |
| *H4 D mac* | 1 | (MH638794)^12^ |
|  | ***1** | **ILC244** |
| *H5 D mac* | 1 | (MH638687)^12^ |
| *H6 D mac* | ***1** | **ILC047** |
| *H7 D mac* | ***1** | **ILC054** |
| *H8 D mac* | ***1** | **ILC224** |
| ***Decapterus macrosoma*** | | |
| *H1 D macro* | 17 | (GU673628)^1^; (HQ560948)^8^; (JF493340-JF493343, JF493346)^4^; (JX261016, JX261033)^8^; (MH638661, MH638663)^12^; (MT609957, MT609958, MT609962-MT609964)^22^; (OL512877)^23^ |
| *H2 D macro* | 4 | (GU673983)^1^, (JX261160)^8^; (MH638795)^12^; (MT609960)^22^ |
| *H3 D macro* | 2 | (JF493344, JF493345)^4^ |
| *H4 D macro* | 2 | (JX261121)^8^; (MT609961)^22^ |
| *H5 D macro* | 1 | (JX261134)^8^ |
|  | ***1** | **ILC144** |
| *H6 D macro* | 1 | (MH638662)^12^, |
| *H7 D macro* | ***6** | **ILC227, ILC228, ILC248, ILCC252, ILC261, ILC265,** |
| *H8 D macro* | ***1** | **ILC264** |
| ***Decapterus muroadsi*** | | |
| *H1 D mur* | 8 | (DSLAR373-08, DSLAR375-08, DSLAR376-08, DSLAR377-08, DSLAR473-09, DSLAR474-09, TZSAL345-13, TZSAL388-13)^4^ |
|  | ***1** | **ILC042** |
| *H2 D mur* | 3 | (FOAF558-07, FOAF559-07, FOAF560-07)^1^ |
| *H3 D mur* | 1 | (TZMSB167-04)^1^ |
| *H4 D mur* | 1 | (LC646723)^13^ |
| ***Naucrates ductor*** | | |
| *H1 N duc* | 4 | (FTWS553-09)^1^; (KU943794)^6^; (KY371779)^5^; (MW379507)^19^ |
|  | ***1** | **ILC110** |
| *H2 N duc* | 2 | (FTWS554-09)^1^; (KU943795)^6^ |
| *H3 N duc* | 1 | (HQ010068)^20^ |
| *H4 N duc* | 1 | (KU943787)^6^ |
| *H5 N duc* | ***1** | **ILC112** |
| ***Selar crumenophthalmus*** | | |
| *H1 S cru* | 28 | (GU673981, GU674083, GU674085, GU674121)^1^; (HQ560967, HQ561001, JX261036, JX261079, JX261080, JX261087, JX261115)^8^; (KJ502064, KJ502066 KJ502069, KJ502074, KJ502077, KJ502079)^25^; (KU943775, KU943791)^6^; (MH085873)^17^; (MH638716, MH638727)^12^; (MW379437, MW379480, MW379500 MW379634, MW379666, MW379785)^19^ |
| *H2 S cru* | 2 | (KJ502071)^25^; (MT772360)^26^ |
|  | ***3** | **ILC234, ILC243, ILC255** |
| *H3 S cru* | 2 | (FJ347941)^24^; KJ502076)^25^ |
| *H4 S cru* | 2 | (HQ560945)^8^; (KJ502062)^25^ |
| *H5 S cru* | 2 | (JX261052)^8^; (KJ502073)^25^ |
| *H 6 S cru* | 2 | (KJ502067)^25^; (MW379575)^19^ |
| *H7 S cru* | 2 | (KJ502075)^25^; (MH085872)^17^ |
| *H8 S cru* | ***1** | **ILC259** |
| ***Selene peruviana*** |  |  |
| *H1 S per* | 5 | (FISHP022-15, FISHP023-15, OXF015-11, RDFCA277-05)^1^; (EU752202)^2^ |
| *H2 S per* | 1 | (HQ974555)^1^; (EU752201)^2^ |
| *H3 S per* | 1 | (FISHP021-15)^1^ |
| *H4 S per* | 1 | (FISHP024-15)^1^ |
| *H5 S per* | 1 | (RDFCA276-05)^1^ |
| *H 6 S per* | 1 | (MF957033)^21^ |
| *H7 S per* | 1 | (MF957034)^21^ |
| *H8 S per* | ***1** | **ILC113** |

**Table S2.** Genetic distances using the Kimura two-parameter model (K2P) based on haplotypes of mitochondrial gene cytochrome c oxidase subunit I (COI) of the nine Carangidae species obtained from reference sequences retrieved from GenBank and BoldSystems and sequences from the present study. Intraspecific variability is in parentheses and bold font. Variability between species of the same genus is shown in shaded areas. *Coryphaena hippurus* (Linnaeus 1758) was used as an outgroup.

|  |  | 1 | 2 | 3 | 4 | 5 | 6 | 7 | 8 | 9 | 10 |
| --- | --- | --- | --- | --- | --- | --- | --- | --- | --- | --- | --- |
| 1 | Outgroup:  *Coryphaena hippurus* | 0.0 |  |  |  |  |  |  |  |  |  |
| 2 | *Selene peruviana* | 23.8 | **(1.34)** |  |  |  |  |  |  |  |  |
| 3 | *Selar crumenophthalmus* | 25.1 | 15.4 | **(0.36)** |  |  |  |  |  |  |  |
| 4 | *Naucrates ductor* | 24.7 | 20.5 | 22.8 | **(0.35)** |  |  |  |  |  |  |
| 5 | *Decapterus muroadsi* | 22.8 | 16.3 | 18.2 | 19.5 | **(0.88)** |  |  |  |  |  |
| 6 | *Decapterus macrosoma* | 24.3 | 16.0 | 17.5 | 19.2 | 6.5 | **(0.53)** |  |  |  |  |
| 7 | *Decapterus macarellus* | 24.2 | 16.4 | 19.0 | 19.6 | 6.4 | 6.4 | **(0.54)** |  |  |  |
| 8 | *Caranx sexfasciatus* | 24.5 | 15.7 | 16.2 | 22.8 | 16.7 | 17.1 | 18.0 | **(0.31)** |  |  |
| 9 | *Caranx caninus* | 24.3 | 16.2 | 17.1 | 20.8 | 16.6 | 17.6 | 18.1 | 8.4 | **(0.29)** |  |
| 10 | *Caranx caballus* | 24.5 | 14.2 | 15.4 | 22.0 | 15.8 | 15.9 | 15.8 | 11.7 | 11.7 | **(0.20)** |

**Table S3.** Systematic list of Carangidae fish species observed or reported (morphologically and molecularly) at Cabo Pulmo National Park (CPNP), located in the southwestern of the Gulf of California, Mexico. Reference number 1 corresponds to species previously listed by Villarreal-Cavazos et al. (2000) and Ayala-Bocos et al. (2018) (from museums/collections, databases, bibliographic references, and field observations of adults); Reference 2, species listed by Ahern et al. (2018) (molecular identification of eggs and larvae); Reference 3, present study (molecular and morphological identification of larvae).

| **Carangidae species reported** | **References** |
| --- | --- |
| *Alectis ciliaris* (Bloch, 1787) | 1 |
| *Ferdauia orthogrammus* (Jordan & Gilbert, 1882) | 2 |
| *Carangoides otrynter* (Jordan & Gilbert, 1883) | 1, 2 |
| *Caranx caballus* Günther, 1868 | 1, 3 |
| *Caranx caninus* Günther, 1867 | 1,2, 3 |
| *Caranx lugubris* Poey, 1860 | 1 |
| *Caranx sexfasciatus* Quoy & Gaimard, 1825 | 1,2, 3 |
| *Decapterus macarellus* (Cuvier, 1833) | 1,2, 3 |
| *Decapterus macrosoma* Bleeker, 1851 | 3 |
| *Decapterus muroadsi* (Temminck & Schlegel, 1844) | 2, 3 |
| *Gnathanodon speciosus* (Forsskål, 1775) | 1 |
| *Elagatis bipinnulata* (Quoy & Gaimard, 1825) | 1 |
| *Naucrates ductor* (Linnaeus, 1758) | 3 |
| *Selar crumenophthalmus* (Bloch, 1793) | 1,2, 3 |
| *Selene brevoortii* (Gill, 1863) | 1 |
| *Selene peruviana* (Guichenot, 1866) | 2, 3 |
| *Seriola lalandi* Valenciennes, 1833 | 1 |
| *Seriola rivoliana* Valenciennes, 1833 | 1,2 |
| *Trachinotus rhodopus* Gill, 1863 | 1, 2 |

References

Ahern ALM, Gómez-Gutiérrez J, Aburto-Oropeza O, Saldierna-Martínez RJ, Johnson AF, Harada AE, Sánchez-Uvera AR, Erisman B, Castro Arvizú DI, Burton RS (2018) DNA sequencing of fish eggs and larvae reveals high species diversity and seasonal changes in spawning activity in the southeastern Gulf of California. Marine Ecology Progress Series 592: 159–179. <https://doi.org/10.3354/meps12446>

Andriyono S, Alam MJ, Kim HW (2020) The Jawa and Bali Island marine fish molecular identification to improve 12S rRNA-tRNA Valin-16S rRNA partial region sequences on the GenBank database. Thalassas: An International Journal of Marine Sciences 36: 343–356. <https://doi.org/10.1007/s41208-020-00196-x>

Asgharian H, Sahafi HH, Ardalan AA, Shekarriz S, Elahi E (2011) Cytochrome c oxidase subunit 1 barcode data of fish of the Nayband National Park in the Persian Gulf and analysis using meta-data flag several cryptic species. Molecular Ecology Resources 11: 461–472. <https://doi.org/10.1111/j.1755-0998.2011.02989.x>

Ayala-Bocos A, Rivera-Melo FJF, Reyes-Bonilla H (2018) Updated checklist of fishes at Cabo Pulmo reef, Gulf of California, Mexico. Revista Ciencias Marinas y Costeras 10: 9–29. <https://doi.org/10.15359/revmar.10-1.1>

Chang CH, Shao KT, Lin HY, Chiu YC, Lee MY, Liu SH, Lin PL (2017) DNA barcodes of the native ray-finned fishes in Taiwan. Molecular Ecology Resources 17: 796–805. <https://doi.org/10.1111/1755-0998.12601>

Dahruddin H, Hutama A, Busson F, Sauri S, Hanner R, Keith P, Hadiaty R, Hubert N (2017) Revisiting the ichthyodiversity of Java and Bali through DNA barcodes: taxonomic coverage, identification accuracy, cryptic diversity and identification of exotic species. Molecular Ecology Resources 17: 288–299. <https://doi.org/10.1111/1755-0998.12528>

Hastings PA, Burton RS (2008) Establishing a DNA sequence database for the marine fish Fauna of California. California Sea Grant College Program, Research Completion Reports. Fisheries, 1–4.

Hou G, Chen WT, Lu HS, Cheng F, Xie SG (2018) Developing a DNA barcode library for perciform fishes in the South China Sea: Species identification, accuracy and cryptic diversity. Molecular Ecology Resources 18: 137–146. <https://doi.org/10.1111/1755-0998.12718>

Hou G, Wang J, Chen Z, Zhou J, Huang W, Zhang H (2020) Molecular and morphological identification and seasonal distribution of eggs of four *Decapterus* fish species in the Northern South China Sea: A Key to conservation of spawning ground. Frontiers in Marine Science 7: 590564. <https://doi.org/10.3389/fmars.2020.590564>

Hou G, Chen Y, Wang S, Wang J, Chen W, Zhang H (2021) Formalin-Fixed Fish Larvae could be effectively identified by DNA Barcodes: A case study on thousands of specimens in South China Sea. Frontiers in Marine Science 8: 634575. <https://doi.org/10.3389/fmars.2021.634575>

Huang D, Chen J, Xu L, Wang X, Ning J, Li Y, Wang L, Liu S, Lin Z, Du F (2022) Larval fish assemblages and distribution patterns in the Zhongsha Atoll (Macclesfield Bank, South China Sea). Frontiers in Marine Science 8: 787765. <https://doi.org/10.3389/fmars.2021.787765>

Hubert N, Meyer CP, Bruggemann HJ, Guerin F, Komeno RJ, Espiau B, Causse R, Williams JT, Planes S (2012) Cryptic diversity in Indo-Pacific coral-reef fishes revealed by DNA-barcoding provides new support to the centre-of-overlap hypothesis. PLOS ONE 7: e28987. <https://doi.org/10.1371/journal.pone.0028987>

Jaonalison H, Durand J-D, Mahafina J, Valade P, Collet A, Cerqueira F, Ponton D (2022) Application of DNA barcoding for monitoring Madagascar fish biodiversity in coastal areas. Diversity 14: 377. <https://doi.org/10.3390/d14050377>

Kimura S, Takeuchi S, Yadome T (2022) Generic revision of the species formerly belonging to the genus *Carangoides* and its related genera (Carangiformes: Carangidae). Ichthyological Research 69: 433–487. <https://doi.org/10.1007/s10228-021-00850-1>

Lakra WS, Verma MS, Goswami M, Lal KK, Mohindra V, Punia P, Gopalakrishnan A, Singh KV, Ward RD, Hebert P (2011) DNA barcoding Indian marine fishes. Molecular Ecology Resources 11: 60–71. <https://doi.org/10.1111/j.1755-0998.2010.02894.x>

Limmon G, Delrieu-Trottin E, Patikawa J, Rijoly F, Dahruddin H, Busson F, Steinke D, Hubert N (2020) Assessing species diversity of Coral Triangle artisanal fisheries: A DNA barcode reference library for the shore fishes retailed at Ambon harbor (Indonesia). Ecology and evolution 10: 3356–3366. <https://doi.org/10.1002/ece3.6128>

Mat Jaafar TNA, Taylor MI, Mohd Nor SA, Bruyn Md, Carvalho GR (2012) DNA barcoding reveals cryptic diversity within commercially exploited Indo-Malay Carangidae (Teleosteii: Perciformes). PLOS ONE 7: 1–16. <https://doi.org/10.1371/journal.pone.0049623>

Mat Jaafar TNA, Taylor MI, Mohd Nor SA, Bruyn Md, Carvalho GR (2020) Comparative genetic stock structure in three species of commercially exploited Indo-Malay Carangidae (Teleosteii, Perciformes). Journal of Fish Biology 96: 337–349. <https://doi.org/10.1111/jfb.14202>

Rathnasuriya MIG, Mateos-Rivera A, Skern-Mauritzen R, Wimalasiri HBU, Jayasinghe RPPK, Krakstad JO, Dalpadado P (2021) Composition and diversity of larval fish in the Indian Ocean using morphological and molecular methods. Marine Biodiversity 51: 39. <https://doi.org/10.1007/s12526-021-01169-w>

Robertson DR, Angulo A, Baldwin C, Pitassy D, Driskell A, Weigt L, Navarro I (2017) Deep-water bony fishes collected by the B/O Miguel Oliver on the shelf edge of Pacific Central America: An annotated, illustrated and DNA-barcoded checklist. Zootaxa 4348: 1–125. <https://doi.org/10.11646/zootaxa.4348.1.1>

Silva-Segundo CA, Funes-Rodríguez R, Gómez-Gutiérrez J, Gallegos-Simental G, Hernández-Trujillo S, Blanco-Jarvio A (2021) DNA barcoding and taxonomic validation of *Caranx* spp. larvae. Journal of the Marine Biological Association of the United Kingdom 101(2): 399–407. <https://doi.org/10.1017/S0025315421000205>

Steinke D, Connell A, Hebert P (2016) Linking adults and immatures of South African marine fishes. Genome 59(11): 959–967. <https://doi.org/10.1139/gen-2015-0212>

Templonuevo RM, Alcantara S, Juanico CS, Yambot A (2018) DNA barcoding of two commercially important fish families (Carangidae and Lutjanidae) collected from Cuyo, Palawan, Philippines. International Journal of Agricultural Technology 14: 2051–2066.

Villarreal-Cavazos A, Reyes-Bonilla H, Bermúdez-Almada B, Arizpe-Covarrubias O (2000) Los peces del arrecife de Cabo Pulmo, Golfo de California, México: lista sistemática y aspectos de abundancia y biogeografía. Revista de Biología Tropical 48(2-3): 413–424.

Ward RD, Holmes BH (2007) An analysis of nucleotide and amino acid variability in the barcode region of cytochrome c oxidase I (cox1) in fishes. Molecular Ecology Notes 7: 899–907. <https://doi.org/10.1111/j.1471-8286.2007.01886.x>

Xu L, Van Damme K, Li H, Ji Y, Wang X, Du F (2019) A molecular approach to the identification of marine fish of the Dongsha Islands (South China Sea). Fisheries Research 213: 105–112. <https://doi.org/10.1016/j.fishres.2019.01.011>

Yancy HF, Zemlak TS, Mason JA, Washington JD, Tenge BJ, Nguyen NL, Barnett JD, Savary WE, Hill WE, Moore MM, Fry FS, Randolph SC, Rogers PL, Hebert PD (2008) Potential use of DNA barcodes in regulatory science: applications of the Regulatory Fish Encyclopedia. Journal of Food Protection 71(1): 210–217. <https://doi.org/10.4315/0362-028X-71.1.210>

Zhang J, Hanner R (2011) DNA barcoding is a useful tool for the identification of marine fishes from Japan. Biochemical Systematics and Ecology 39: 31–42. <https://doi.org/10.1016/j.bse.2010.12.017>
